# Prophage Gifsy-1 Induction in Salmonella enterica Serovar Typhimurium Reduces Persister Cell Formation after Ciprofloxacin Exposure

**DOI:** 10.1128/spectrum.01874-23

**Published:** 2023-06-12

**Authors:** Sebastian Braetz, Peter Schwerk, Nara Figueroa-Bossi, Karsten Tedin, Marcus Fulde

**Affiliations:** a Institute of Microbiology and Epizootics, Freie Universität Berlin, Berlin, Germany; b Veterinary Centre for Resistance Research (TZR), Freie Universität Berlin, Berlin, Germany; c Université Paris-Saclay, CEA, CNRS, Institute for Integrative Biology of the Cell (I2BC), Gif-sur-Yvette, France; University of Wollongong

**Keywords:** Gifsy-1, *Salmonella*, bacteriophage, persister cells

## Abstract

Persister cells are drug-tolerant bacteria capable of surviving antibiotic treatment despite the absence of heritable resistance mechanisms. It is generally thought that persister cells survive antibiotic exposure through the implementation of stress responses and/or energy-sparing strategies. Exposure to DNA gyrase-targeting antibiotics could be particularly detrimental for bacteria that carry prophages integrated in their genomes. Gyrase inhibitors are known to induce prophages to switch from their dormant lysogenic state into the lytic cycle, causing the lysis of their bacterial host. However, the influence of resident prophages on the formation of persister cells has only been recently appreciated. Here, we evaluated the effect of endogenous prophage carriage on the generation of bacterial persistence during Salmonella enterica serovar Typhimurium exposure to both gyrase-targeting antibiotics and other classes of bactericidal antibiotics. Results from the analysis of strain variants harboring different prophage combinations revealed that prophages play a major role in limiting the formation of persister cells during exposure to DNA-damaging antibiotics. In particular, we present evidence that prophage Gifsy-1 (and its encoded lysis proteins) are major factors limiting persister cell formation upon ciprofloxacin exposure. Resident prophages also appear to have a significant impact on the initial drug susceptibility, resulting in an alteration of the characteristic biphasic killing curve of persister cells into a triphasic curve. In contrast, a prophage-free derivative of *S.* Typhimurium showed no difference in the killing kinetics for β-lactam or aminoglycoside antibiotics. Our study demonstrates that induction of prophages increased the susceptibility toward DNA gyrase inhibitors in *S.* Typhimurium, suggesting that prophages have the potential for enhancing antibiotic efficacy.

**IMPORTANCE** Bacterial infections resulting from antibiotic treatment failure can often be traced to nonresistant persister cells. Moreover, intermittent or single treatment of persister cells with β-lactam antibiotics or fluoroquinolones can lead to the formation of drug-resistant bacteria and to the emergence of multiresistant strains. It is therefore important to have a better understanding of the mechanisms that impact persister formation. Our results indicate that prophage-associated bacterial killing significantly reduces persister cell formation in lysogenic cells exposed to DNA-gyrase-targeting drugs. This suggests that therapies based on gyrase inhibitors should be favored over alternative strategies when dealing with lysogenic pathogens.

## INTRODUCTION

Bacterial persister cells are generally considered to be a slow-growing, metabolically inactive subpopulation with high tolerance to otherwise lethal concentrations of bactericidal antibiotics and which can be responsible for recalcitrant infections (1, 2). Persistence is a phenotypic variant of resistance; however, in contrast to resistant and heteroresistant bacteria, persister cells do not proliferate in the presence of antibiotics (1). In general, persister cells represent only a small subpopulation of a bacterial culture, generally less than 0.1% of a growing culture. Persistence is observed in killing assays in which the majority of the drug-sensitive bacteria are rapidly killed with increasing time of treatment until only a plateau of bacterial survivors (persister cells) remain. This rapid killing of drug-sensitive bacteria results in a biphasic killing curve, with a rapid decline of viable cells during an initial phase followed by a second phase characterized by slow rates of killing, representing the phenotypically resistant persister cells (3). Currently, there are two hypotheses as to how bacterial persister cells become established: stochastically during the exponential growth phase and triggered or induced by stress (3). Numerous factors have been reported which can have an influence on persister cell formation, including the involvement of efflux pumps, oxidative stress, energy (ATP) levels, toxin-antitoxin modules, protein aggregation, or the SOS response (4–10). However, the impact on persister cell formation by genes or products encoded by endogenous prophages is less well understood, although prophages are often considered a natural part of bacterial DNA (11).

Prior studies have reported both beneficial and adverse effects of endogenous prophages on bacterial survival after antibiotic exposure (12–14). For example, Sandvik et al. concluded that phage induction was involved in the maximal killing of Staphylococcus aureus by ciprofloxacin, suggesting that resident prophage enhanced antibiotic susceptibility (14). In another study, it was found that deletion of 9 cryptic prophages in Escherichia coli increased antibiotic susceptibility, including quinolone antibiotics, indicating beneficial effects of prophages toward antibiotic resistance (12). More recently, it was found that both endogenous cryptic prophages and E. coli K-12 strains infected and lysogenized with the lambdoid phage φ80 resulted in increased killing of antibiotic-tolerant persistent bacteria due to induction of the prophage by antibiotics (15). These prior studies suggest that the role of endogenous prophages in bacterial susceptibility to antibiotics can be influenced by the status of endogenous prophages, e.g., whether cryptic or inducible, or by prophage gene expression in the absence of active phage replication.

Salmonella enterica serovar Typhimurium harbors a variable assortment of prophages, including Fels-1, Fels-2, Gifsy-1, Gifsy-2, Gifsy-3, and ST64B (16–18). These temperate prophages generally remain dormant as lysogens, integrated into the chromosome of the host. Activation of the prophages to switch from the lysogenic to the lytic cycle occurs as part of the SOS response, which can be triggered when bacteria are exposed to DNA-damaging agents such as mitomycin C or drugs that block DNA replication (e.g., DNA gyrase inhibitors) (19, 20). In a temporally regulated manner, after activation leading to excision of the prophages from the chromosome, the expression of lysis proteins is also induced as part of the late gene expression. In the lambdoid phages such as bacteriophage λ in E. coli, host cell lysis occurs after expression of the pore-forming holin (*S*) proteins, the cell wall-degrading endolysin (*R*), and the auxiliary spanins (*RzRz1*) at the end of the lytic cycle between 50 and 60 min postinduction, resulting in phage release (21).

In this study, we investigated the impact of the four resident prophages of *S.* Typhimurium strain ATCC 14028s on antibiotic susceptibility to the bactericidal fluoroquinolone antibiotic ciprofloxacin. We show that deletion of the four endogenous prophages in this *S.* Typhimurium strain changes the biphasic killing curve which is characteristic for persister cell formation into a triphasic killing curve characterized by a delayed entry into the killing phase. The results indicate that Gifsy-1 has a significant role in the initial killing and final levels of surviving bacteria after ciprofloxacin treatment, where reduced survival was attributed to the expression of the gene homologues of the bacteriophage λ lysis proteins S, R, and RzRz1 (22). In contrast, the contribution of Gifsy-2, Gifsy-3, and ST64B to killing by ciprofloxacin was negligible.

## RESULTS

### Gifsy-1 lysis proteins reduce persister cell formation.

**FIG 1:**
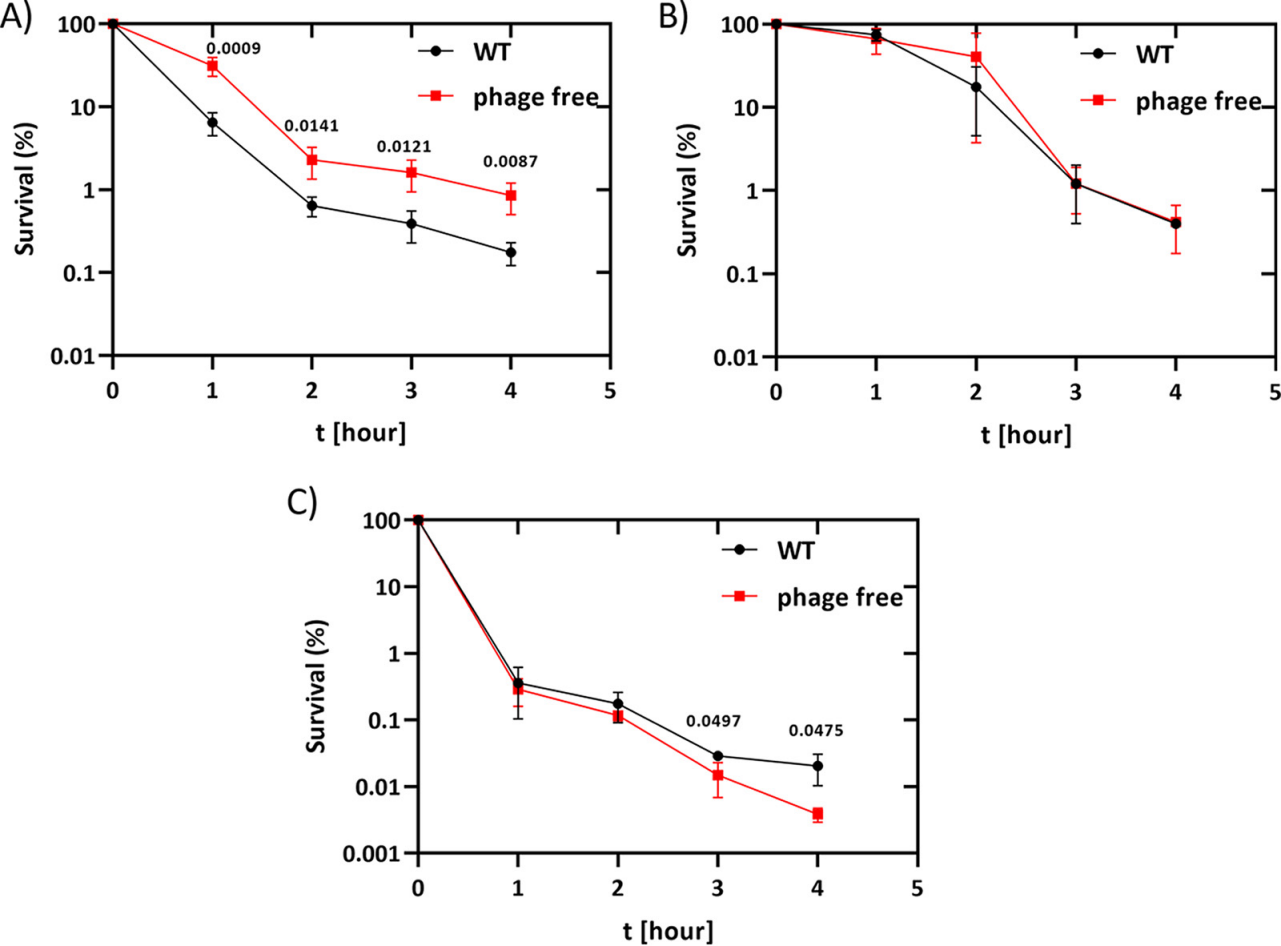
Persister assays with ciprofloxacin, carbenicillin, and kanamycin. (A to C) *S.* Typhimurium 14028s (8640) and its prophage-free variant (11126) were treated with (A) ciprofloxacin, (B) carbenicillin, and (C) kanamycin with 4-fold MIC, and survival was determined at the indicated time points by plating the bacteria on LB plates. Persister assays were performed at least three times, and significance was calculated using the unpaired two-tailed Student’s *t* test.

**FIG 2:**
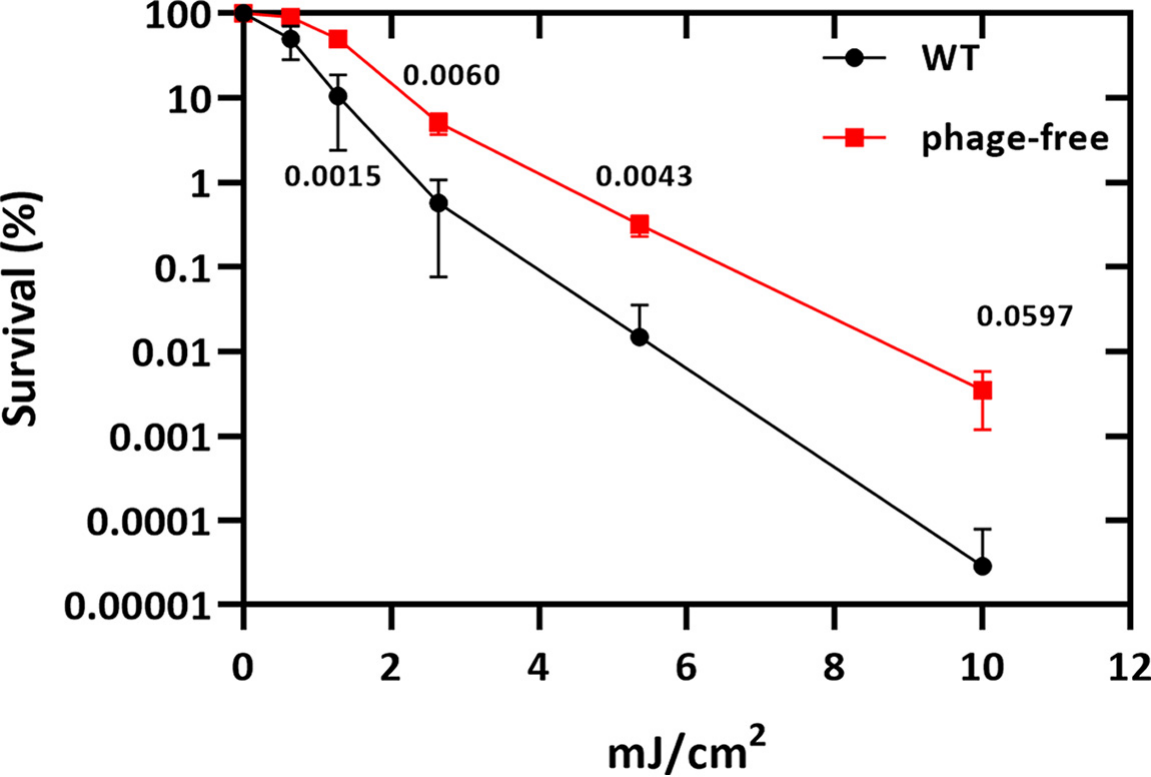
UV survival assay. Both strains, *S.* Typhimurium (8640) and the phage-free variant (11126), were incubated to the mid-log phase before being exposed to UV. Experiments were conducted at least three times, and significance was calculated using the unpaired two-tailored Student’s *t* test.

In order to investigate a possible role for the endogenous prophage in killing and persistence toward bactericidal antibiotics, we chose to work with *S.* Typhimurium ATCC 14028, a well-characterized strain used in both *in vitro* and *in vivo* virulence and infection models (18, 20). To investigate the role of prophages in antibiotic tolerance and/or persistence, we generated derivatives of *S.* Typhimurium ATCC 14028 in which the resident prophages were sequentially deleted from the chromosome (see Table S1 in the supplemental material). We then compared the survival of the wild-type with a prophage-free variant of *S.* Typhimurium ATCC 14028s after a challenge with the gyrase inhibitor ciprofloxacin (1 μg/mL) at the indicated time points. As shown in Fig. 1A, deletion of all four prophages significantly delayed the initial killing phase and increased persister cell formation approximately 3- to 5-fold compared to the wild-type strain harboring all four prophages. These results were very similar to the effects of UV irradiation, where the prophage-free strain was also more tolerant to UV than the wild type (Fig. 2). In contrast, deletion of the prophages did not affect the killing kinetics or formation of persister cells after treatments with either carbenicillin or kanamycin (Fig. 1B and C). These results were consistent with the DNA-damaging effects of both UV irradiation and gyrase inhibitors, leading to induction of the SOS response and phage induction (23–26).

As the initial killing kinetics appeared to be reduced and the final levels of persister cells were higher in the prophage deletion strain, we examined the killing kinetics after addition of ciprofloxacin more closely. The initial slope of the killing curve of the prophage-free variant indicated a delay during the first hour post-ciprofloxacin challenge compared to the wild-type strain, leading to a triphasic killing curve (Fig. 1A). After 1 h of treatment, the slope of the killing curves of both strains were comparable again for up to 2 h posttreatment. However, the final plateau of surviving bacteria also appeared to show the same kinetics, i.e., the appearance of persister cells occurred in both strains at approximately 2 h postchallenge, resulting in more viable bacteria for the prophage-free strain and a significantly increased persister cell population after entry into the plateau phase (Fig. 1A). These results suggested that a large fraction of the bacterial killing during the first hour of ciprofloxacin treatment was dependent on the endogenous prophage.

**FIG 3:**
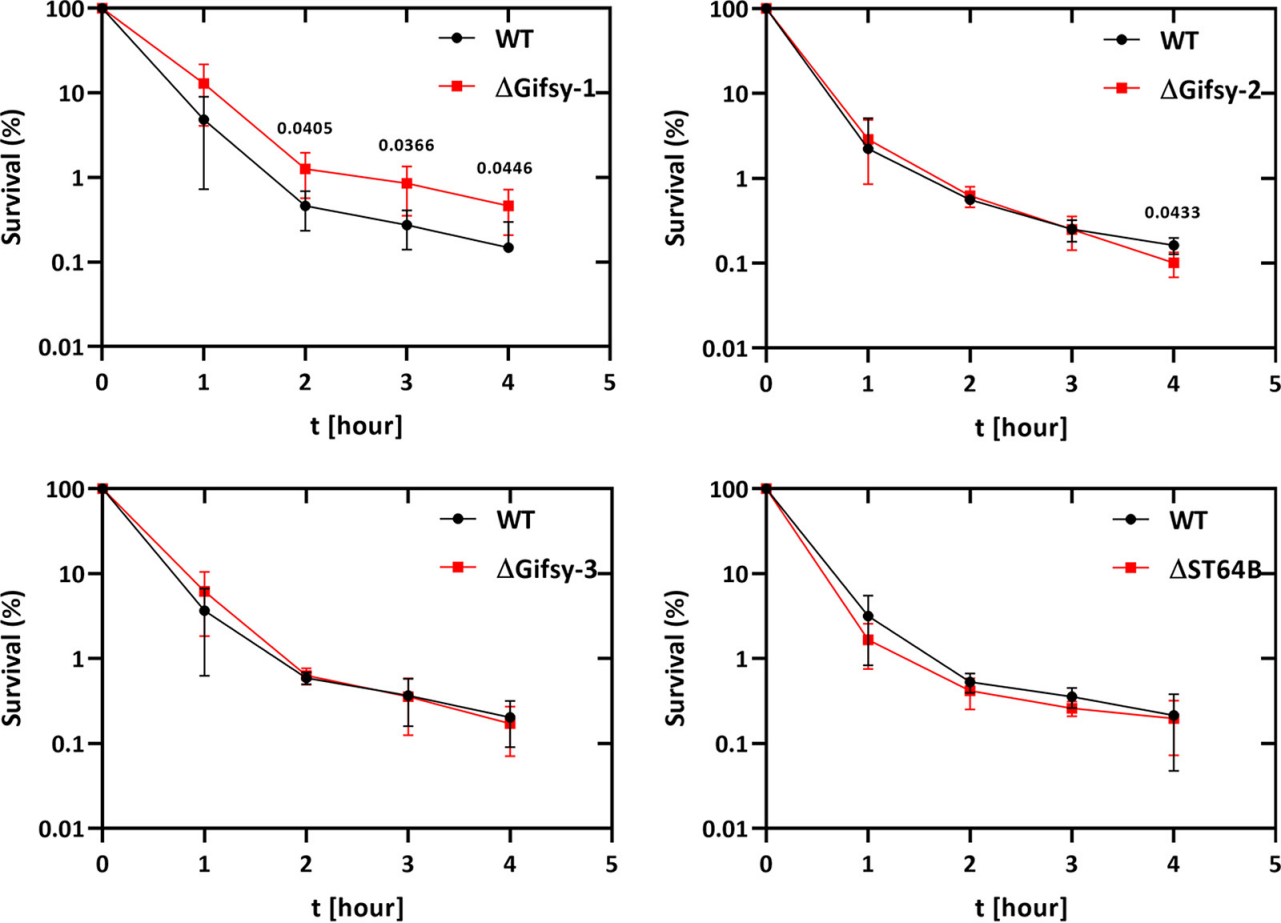
Persister assays with single prophage deletions. The Salmonella wild type 14028s (8640) and the corresponding single prophage deletion mutants (11160 = ΔGifsy-1; 11162 = ΔGifsy-2; 11224 = ΔGifsy-3; 10926 = ΔST64B) were incubated to the mid-exponential phase before being challenged with 4-fold the MIC of ciprofloxacin. At least three independent experiments were performed, and significance was calculated using the unpaired two-tailed Student’s *t* test.

To investigate the involvement of the phages in more detail, we also performed persister assays after ciprofloxacin challenge with strains harboring deletions of the individual prophages. Only the strain harboring a deletion of Gifsy-1 showed a reduction in the killing kinetics and increased persistence similar to that of the strain deleted for all four prophages relative to the wild-type strain (Fig. 3). In contrast, the contribution of Gifsy-2, Gifsy-3, and ST64B to survival of Salmonella was negligible (Fig. 3). To determine the level of induction for the four endogenous prophages, Gifsy-1, -2, and -3, and ST64B, we performed real-time quantitative PCR (qRT-PCR) to examine the induction kinetics of each of the prophages after ciprofloxacin challenge. Real-time PCR confirmed that among the four prophages, Gifsy-1 showed the highest level of induction, whereas induction of Gifsy-2 and Gifsy-3 was more modest (Fig. S1). We were unable to detect a signal for ST64B in these assays (data not shown).

**FIG 4:**
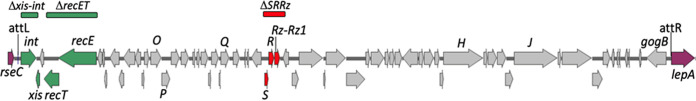
Schematic diagram of the Gifsy-1 prophage showing the positions of the relevant genes and corresponding deletions (overlying bars) analyzed in this study. Single capital letters denote genes named on the basis of their similarity to genes in bacteriophage λ.

**FIG 5:**
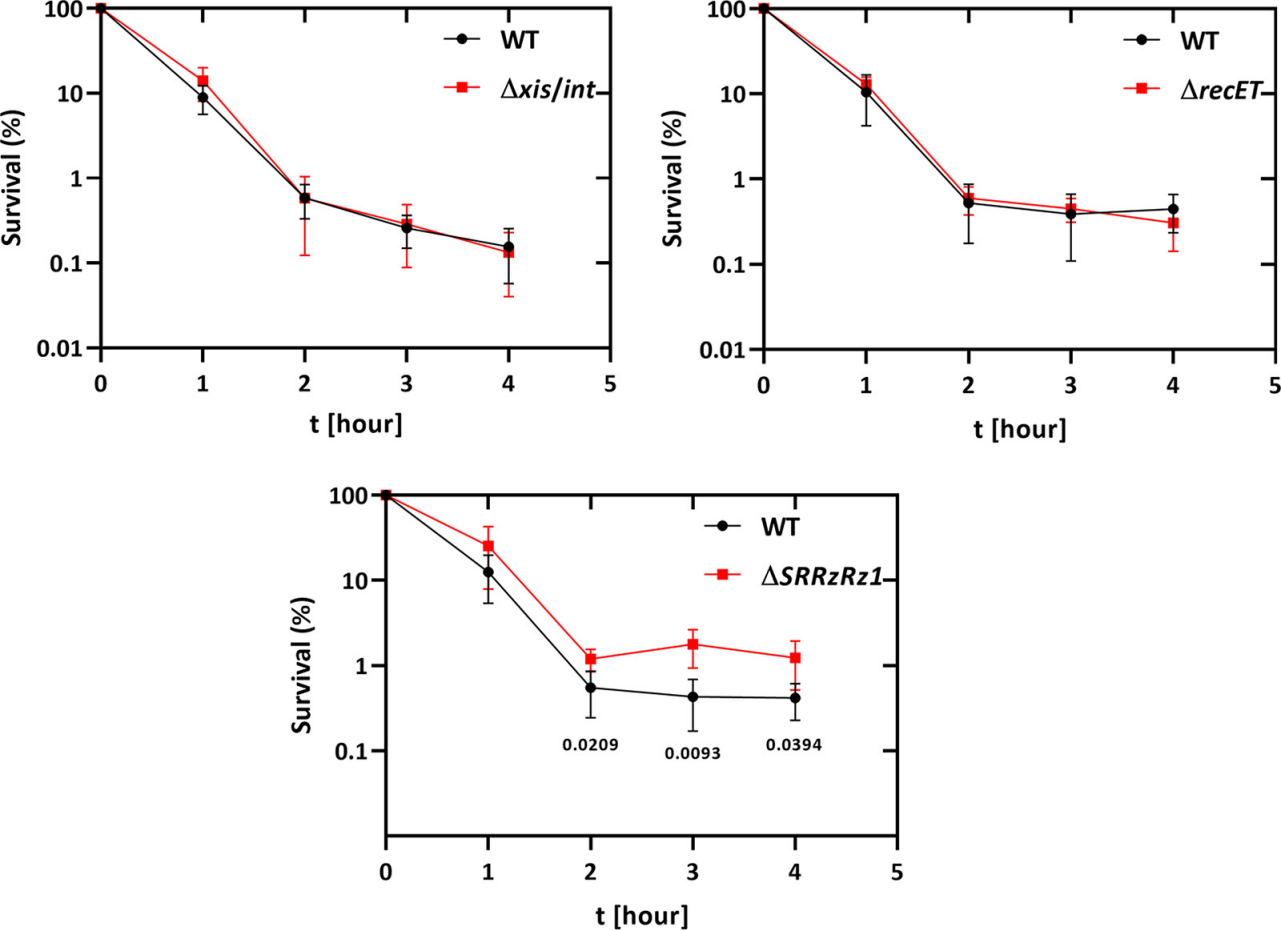
Persister assay with strains harboring specific gene deletions within Gifsy-1. The corresponding strains (8640 = *S. Typhimurium* wild type; 11976 = Δ*recET*; 11958 = Δ*xis*/*int*; 12074 = Δ*SRRzRz1*) were incubated to the mid-log phase before treatment with 1 μg/mL ciprofloxacin. The genes *xis*/*int* are responsible for the integration and excision of Gifsy-1 from the bacterial chromosome, while *recET* catalyzes homolog recombination. The bacteriophage λ gene homologues S, R, and RzRz1 are responsible for the lysis of their host. Experiments were conducted at least three times, and significance was calculated using the unpaired two-tailored Student’s *t* test.

The preceding results suggested that induction of the lytic cycle of the prophage Gifsy-1 in *S. Typhimurium* contributed to the killing kinetics and final persister cell population after ciprofloxacin treatment. In order to understand the mechanisms behind the apparent Gifsy-1-dependent effects on killing and persistence, we tested various gene deletions within Gifsy-1 to determine which prophage functions might play a role. One of the earliest events in phage induction and a requirement for viral genome replication is the excision of the phage genome out of the bacterial chromosome. We therefore repeated the ciprofloxacin killing and persister assays using derivatives of Gifsy-1 harboring deletions of the integrase/excisionase (*int*/*xis*) genes, involved in the excision of the phage from the host chromosome (27), and the *recET* genes encoding the bacteriophage recombination proteins RecET (Fig. 4). As the recombination proteins RecFOR of E. coli have been proposed to bias the induction of the SOS response (28), it was therefore possible that the bacteriophage recombination proteins might show a similar effect. In addition, we also examined the role of the lysis proteins S, R, and RzRz1, which are active during the last phase of prophage induction and are responsible for host cell lysis and phage release (21, 29) (Fig. 4). As seen in Fig. 5, only deletion of the Gifsy-1 homologs of the bacteriophage λ *S*, *R*, and *Rz* lysis genes (annotated STM14_3196, STM14_3195, and STM14_3194, respectively, in the *S.* Typhimurium ATCC 14028 genome [22]) showed the same increased persister cell formation after ciprofloxacin treatment as seen for the prophage-free (Fig. 1A) and ΔGifsy-1 deletion strains (Fig. 3). Neither loss of excision/integrase functions in the Gifsy-1 Δ*xis-int* prophage nor the deletion of *recET* significantly affected persister cell formation (Fig. 5). Furthermore, the strain harboring the Gifsy-1 Δ*SRRz* homolog deletions showed up to 10-fold higher levels of surviving bacteria (persisters) after exposure to ciprofloxacin at 24 h postchallenge, supporting the idea that the endogenous Gifsy-1 prophage plays a significant role in both the formation and magnitude of the final persister cell population surviving a challenge with ciprofloxacin (Fig. S2).

## DISCUSSION

This study demonstrated that the endogenous prophages of *S.* Typhimurium can have a significant impact on antibiotic tolerance and persistence toward bactericidal antibiotics. Both UV irradiation and ciprofloxacin challenge resulted in up to 10-fold higher levels of bacterial survivors in the *S.* Typhimurium strain deleted for the four endogenous prophages. In particular, the induction of the prophages during the initial killing phase during the first hour postchallenge played a crucial role, as seen by the delayed onset of killing in the prophage-free variant compared to the wild-type strain (Fig. 1A). This observation strongly suggests that the fluoroquinolone-mediated killing mechanism is the same in both strains and differs only in the additional host lysis events due to induction of prophages in the wild-type strain. In the absence of the endogenous prophage, up to 10-fold more bacteria survived the initial killing and contributed to the final persister cell population. That the difference in the killing kinetics and final levels of persister cells results from prophage induction is most clearly shown by comparing the persister cell levels between the wild-type and the Gifsy-1 strain after deletion of its lysis genes (Fig. 3 and Fig. 5). Here, loss of the expression of the holin, endolysin, and spanin significantly increased persister cell formation after ciprofloxacin treatment, indicating that other phage functions do not appear to influence the generation of persister cells. These results also indicate that Gifsy-1 does not need to be excised from its host chromosome to initiate lysis since the killing curve of the Gifsy-1 Δ*xis*/*int* mutant, which remains integrated in the bacterial chromosome, was indistinguishable from the wild type (Fig. 5). These results are also consistent with earlier observations in E. coli for bacteriophage λ *int* lysogens, which show severe reductions in phage burst size but nevertheless result in host cell killing and lysis after induction, despite the inability for phage excision (30, 31).

These observations may explain discrepancies between our observations regarding reduced killing and increased persister cell formation in the phage-free and ΔGifsy-1 strains and the results of Wang et al., who found that the endogenous, cryptic phages of E. coli conferred resistance to quinolone antibiotics (12). In their study of an E. coli strain deleted for all 9 cryptic prophages (referred to as the Δ9 strain), the authors describe the E. coli cryptic phage as inactive in terms of cell lysis. In addition, at least two of the cryptic phages (CP4-6 and Rac) were found to express inhibitors of cell division involved in antibiotic resistance (12). Therefore, it is likely that the cryptic phages described in the study of Wang et al. had lost their lytic functions, rendering them permanently inactive with regard to host cell lysis. In addition, the prophages e14, CP4-6, and Rac showed low levels of excision under normal growth conditions, but only e14 was inducible by mitomycin C addition or oxidative stress.

On the other hand, our study is consistent with the observations of Harms et al. in which strains of E. coli harboring lysogens of the lambdoid phage φ80 were found to be more sensitive to killing and showed reduced persisters in response to ciprofloxacin (15). φ80 (also known as Lula) is a temperate lambdoid phage which is easily triggered to induction by DNA damage (32). The role of the inducible φ80 prophage in antibiotic resistance and persistence was discovered inadvertently following a study of the role of toxin-antitoxin (TA) systems in antibiotic tolerance and persistence in E. coli (33). The authors of that study found that successive deletion of 10 of the toxin-antitoxin systems present in the E. coli genome (Δ10 strain) reduced the persistence of the strains against the antibiotics ciprofloxacin and ampicillin, consistent with a role for toxin-antitoxin systems in bacterial persistence against bactericidal antibiotics. Further studies by this group, however, revealed that many of the TA deletion strains had been lysogenized by φ80, which was induced by the antibiotic treatments used to determine the effects of TA gene deletions (15). Although the φ80 infections and lysogenization of experimental strains led to unfortunate misinterpretation of results regarding the TA systems (33), we suggest the findings of the follow-up studies investigating the role of prophage induction on persistence to antibiotics (15) is an equally, if not more, important observation.

It has been previously observed that high doses of bactericidal antibiotics, including quinolones and fluoroquinolones, are less efficient in bacterial killing and yield higher levels of surviving persister cells (34, 35). This observation has been partially explained by inhibition of RNA and protein synthesis at high concentrations of antibiotics. We suggest that an additional effect involves the induction of endogenous prophages, which also require host transcription and translation activities for induction, synthesis, and phage assembly (17). Inhibition of translation could also promote persister cell formation by interfering with phage induction, which synergistically contributes to the lysis of the host (21, 29).

The results of our study demonstrated that endogenous phages have the potential to increase the efficiency of DNA-damaging agents, consistent with previous studies in which a nonlysogenic strain of E. coli for the phage lambda λ was also more tolerant against danofloxacin than the lysogenic counterpart (13). These results are reminiscent of a phenomenon called phage-antibiotic synergy (PAS), in which sublethal concentrations of antibiotics (β-lactam and fluoroquinolone) increased phage production and phage-mediated burst size (36). This was later attributed to a delayed lysis of the host allowing prolonged assembly and higher numbers of infectious phages (37). This suggests that phages can be used as natural adjuvants to increase the efficiency of antibiotics. However, the prerequisite is that the bacteria must have their own prophages integrated in the bacterial chromosome.

Bacteriophages were considered for use to treat bacterial infections long before the discovery of penicillin and later during the cold war in eastern Europe (38). Due to the worldwide increase in antibiotic resistance, there has been renewed interest in bacteriophages as an alternative to antibiotics to treat bacterial infections (39). In recent years, several promising approaches have been reported for using phages to kill pathogens, including the usage of phage cocktails or purified phage proteins (holins/endolysins), a combinatorial treatment with phages and antibiotics, or application of bioengineered phages as vaccines (40). Lysogenic as well as lytic phages could therefore offer new strategies to eradicate drug-persistent bacteria as natural antibacterial agents or increase the efficiency of available antibiotics.

## MATERIALS AND METHODS

### Media and antibiotics.

Lysogeny broth (LB Lennox, Carl Roth) was used for growth of bacterial cultures. Where appropriate for selection or screening, the antibiotics kanamycin (50 μg/mL), carbenicillin (100 μg/mL), and chloramphenicol (15 μg/mL) (Carl Roth) were added to either liquid or solid agar medium. The MICs for the strains were determined for kanamycin (3.12 μg/mL), carbenicillin (3.12 μg/mL), and ciprofloxacin (0.125 μg/mL; Sigma-Aldrich) in microplate assays according to CLSI recommendations. Standard MIC assays are performed in 1:1 dilution steps. Here, the designation 4× MIC refers to the number of dilution steps beyond the determined MIC value rather than 4-fold the determined MIC. For our strain, the MIC for ciprofloxacin was determined as 0.125 μg/mL. Four dilution steps back from this value in the MIC assays starting from the MIC of 0.125 μg/mL corresponds to 0.125 (1× MIC), 0.25 (2× MIC), 0.5 (3× MIC), and 1.0 μg/mL (4× MIC). For persister assays, cultures were treated with the indicated antibiotics at 4-fold the MIC.

### Bacterial strains.

All strains used in this study are summarized in Table S1, including the original strains for P22 lysate production. The strain background used in this study is Salmonella enterica serovar Typhimurium strain ATCC 14028s harboring a *gyrA*(D87Y) mutation conferring nalidixin resistance. Note that while the *gyrA* mutation increases the MIC for ciprofloxacin, it does not affect the killing kinetics (41, 42). The *gyrA* strain background was chosen to avoid accumulation of primary *gyrA* mutations, which occur relatively frequently (43, 44) and which would grow overnight in the persister assays which were carried out for up to 24 h.

The strains harboring deletions of the Gifsy-1 (ΔGifsy-2::*kan*), Gifsy-2 (ΔGifsy-2::*cat*), and ST64B (ΔST64B::*kan*) prophage have previously been described (45). Construction of the ΔGifsy-3::*kan* deletion strain (MA14253) was performed using the λ Red mutagenesis/gene replacement method (46). Briefly, the primers ppAG72 and ppAG73 containing homologous sequences upstream and downstream of the Gifsy-3 integration site in the *S.* Typhimurium *icdA* gene were used to PCR amplify the kanamycin resistance cassette in plasmid pKD13 (46). The primers were designed to regenerate a wild-type *icdA* gene sequence after recombination, which would otherwise be lost when generating a full deletion of Gifsy-3. The purified PCR product was then used to transform competent cells which had been induced for expression of the bacteriophage λ Red recombinase as previously described (46), with selection for kanamycin resistance. Putative recombinants were screened by PCR using the primer pairs pp599-ppR25 and pp587-pp549 to verify the left and right recombination junctions, respectively. Isolates which were positive in the PCR screening were further sequenced to verify the deletion of Gifsy-3 from the chromosome. The resulting strain, MA14253, harboring the ΔGifsy-3::*kan* deletion/replacement was then used as a donor for P22 transductions into other strains using standard protocols. Additional gene deletions of the Gifsy-1 endoloysin homologs (Δ*SRRz*::*kan*), recombinase (Δ*recET*::*kan*), and excision/integrase genes (Δ*xis-int*::*kan*) were constructed in a similar manner, but using the kanamycin resistance cassette of pKD4 (46). All primers used in this work are listed in Table S3.

To construct the prophage-free strain, phage P22 lysates were first prepared on donor strains harboring deletions of the respective prophage in which the prophage had been replaced with a kanamycin or chloramphenicol resistance cassette (see Table S1). The phage deletions were introduced into our wild-type background (8640) using bacteriophage P22 transductions followed by plating on the appropriate antibiotic selection plates according to standard protocols. Putative transductants were then purified as single colonies on Green plates to avoid carryover of infectious P22 (47) and screened for deletion of the respective prophage by PCR. Isolates harboring the correct prophage deletion were then transformed with the FLP-recombinase plasmid pCP20 to eliminate the antibiotic resistance cassette (46, 48). After screening for loss of kanamycin or chloramphenicol resistance, plasmid pCP20 was subsequently eliminated by growth at the nonpermissive temperature (37°C) for plasmid replication and screened for loss of ampicillin and chloramphenicol resistance at the permissive temperature (30°C) (46). The strain harboring a deletion of one of the prophage was then used as a host for P22 transduction using lysates prepared on a second prophage deletion strain, and the procedure as described above was repeated.

### Persister assays and UV killing.

Strains for the persister assays were streaked out to Lennox agar plates and incubated overnight at 37°C. The following day, a single colony was used to inoculate 6 mL of Lennox broth in a 16 by 160-mm glass culture tube and were incubated at 37°C with shaking (200 rpm) in a fixed rack with tubes at an angle of approximately 45° to ensure aeration. At an optical density at 600 nm (OD_600_) of 0.5, the CFU/mL of each culture was adjusted to approximately 5 × 10^6^/mL for the persister assays, or bacterial suspensions were adjusted to an optical density (OD_600_) of 0.01 for UV killing in 6 mL Lennox broth. For the persister assays, ciprofloxacin (1 μg/mL), kanamycin (25 μg/mL), and carbenicillin (25 μg/mL) were used at 4-fold (4×) the MIC as described above. After addition of the antibiotics, the cultures were further incubated at 37°C with aeration. The bacterial survival and persistence was determined by removing aliquots at the time points indicated in the figures. The aliquots were centrifuged, and the bacterial pellets were resuspended in 1× PBS and plated on Lennox agar plates for colony counting. For UV killing assays, the bacterial suspensions were transferred to a petri dish and exposed to UV light using a mercury vapor discharge lamp (50 W/m^2^). After irradiation, the bacteria were plated on LB plates and incubated overnight at 37°C. All assays were conducted at least three times, independently.

### Real-time PCR (qPCR).

The wild type was incubated to the mid-log phase as described above and subsequently treated with 1 μg/mL ciprofloxacin for 60 min. After treatment, the RNA was extracted using the RNeasy minikit (Qiagen) according to the manufacturer’ recommendations. The extracted RNA was transcribed into 1,000 ng cDNA as follows: a reaction mixture containing 2 μL of 50 μM oligo(dT)18 (Eurofins Genomics), 2 μL of 10 mM deoxynucleoside triphosphates (dNTPs; Invitrogen), 1 μL of 200 U/μL reverse transcriptase (Thermo Scientific), and 0.5 μL of 40 U/μL RiboLock RNase inhibitor (Thermo Scientific) was brought to a final volume of 20 μL with sterile water. Subsequently, 25 ng/well cDNA was used to measure the expression of the phage genes, using 0.2 μL of 10 pmol/μL primers from Table S2, and mixed with 10 μL (10×) SYBR green master mix (Thermo Fisher). The fluorescence signal was determined using an Applied Biosystems StepOnePlus device, using the following protocol: denaturation at 95°C for 2 min, elongation at 60°C for 30 sec, and denaturation at 95°C for 10 sec (cycle 40×). After 40 cycles, elongation was carried out at 60°C for 1 min followed by the final denaturation step at 95°C for 15 sec and the final elongation step at 60°C for 1 min.
