# Supplementary material for: Prophage Gifsy-1 Induction in Salmonella enterica Serovar Typhimurium Reduces Persister Cell Formation after Ciprofloxacin Exposure
Source: Microbiol Spectr. 2023 Jun 12;11(4):e01874-23. doi: 10.1128/spectrum.01874-23 (PMC10433948; doi:10.1128/spectrum.01874-23)
Supplement: Supplemental file 1 — Fig. S1 and S2. Download spectrum.01874-23-s0001.pdf, PDF file, 0.1 MB [file spectrum.01874-23-s0001.pdf]

A) B)

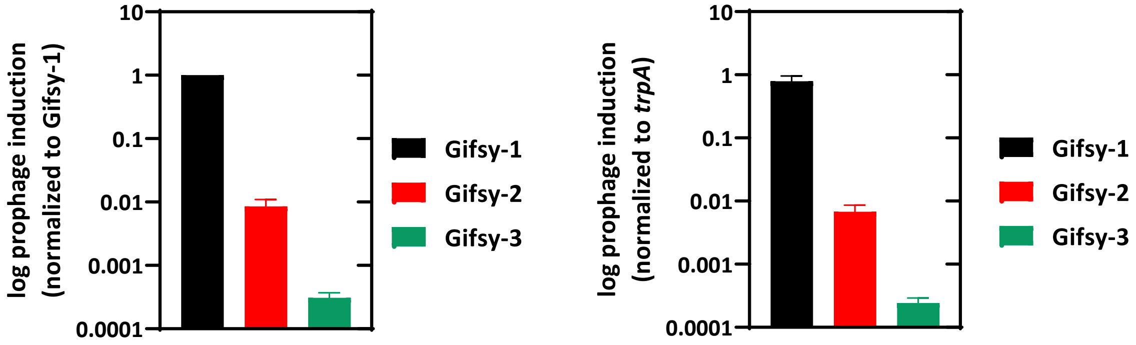

Supp. Fig. 1 Only Gifsy-1 is significantly induced by ciprofloxacin treatment. The wild-type strain was exposed for 60 min to ciprofloxacin and phage induction was measured via real time PCR. The expression of the phage genes was normalized to the household gene *trpA*. (A) The prophage induction levels of Gifsy-2, and -3 normalized to the induction levels of Gifsy-1. (B) The relative prophage induction levels of Gifsy-1, -2, and -3 compared to the bacterial *trpA* gene expression. The assays were performed three times, independently in triplicate. The data shown is representative of one experiment.

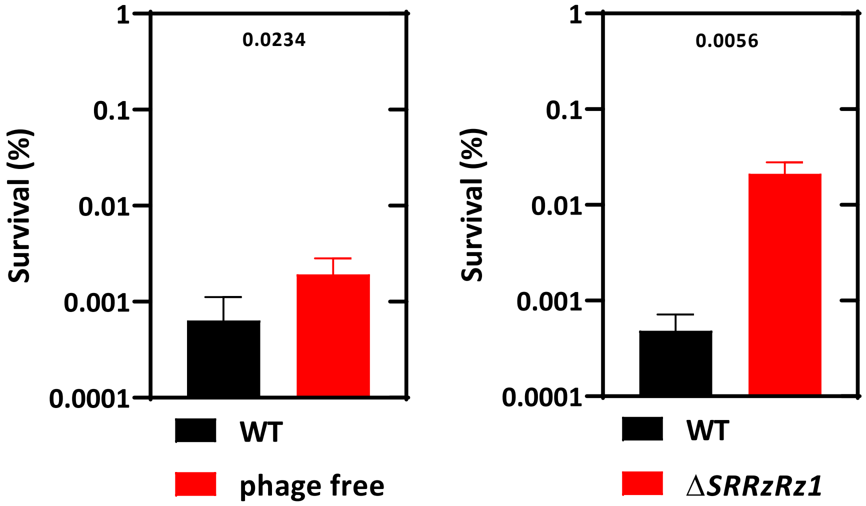

Supp. Fig. 2 Survival after 24 hours of treatment with ciprofloxacin. The *Salmonella* wild-type 14028s (8640), phage-free variant (left panel) and the Gifsy-1 lysis genes Δ*SRRzRz1* deletion mutant (right panel) were grown to mid-log phase growth phase and subsequently exposed to four-fold the MIC of ciprofloxacin for 24 hours. The data shown are from at least three, independent experiments, and significance was calculated using an unpaired, two-tailed Student`s t-test.
