# Supplementary material for: Prophage Gifsy-1 Induction in Salmonella enterica Serovar Typhimurium Reduces Persister Cell Formation after Ciprofloxacin Exposure
Source: Microbiol Spectr. 2023 Jun 12;11(4):e01874-23. doi: 10.1128/spectrum.01874-23 (PMC10433948; doi:10.1128/spectrum.01874-23)
Supplement: Supplemental file 2 — Tables S1 to S3. Download spectrum.01874-23-s0002.docx, DOCX file, 0.02 MB [file spectrum.01874-23-s0002.docx]

**Supplementary data**

| **Table S1**: Strains used in this study | | |
| --- | --- | --- |
| Strain | Relevant Features* | Source |
| JS215 | *S.* Typhimurium ATCC 14028 ΔGifsy-1::*kan* | [1] |
| JS226 | *S.* Typhimurium ATCC 14028 ΔGifsy-2::*cat* | [2] |
| MA7549 | *S.* Typhimurium ATCC 14028 ΔGifsy-1 ΔGifsy-2 ΔGifsy-2 ΔST64B::*kan* | [3] |
| MA14253 | *S.* Typhimurium ATCC 14028 ΔGifsy-3::*kan* | This study |
| 8640 | *S.* Typhimurium ATCC 14028 NalR *gyrA*(D87Y) | [4] |
| 10926 | *S.* Typhimurium ATCC 14028 NalR ΔST64B | This study |
| 11126 | *S.* Typhimurium ATCC 14028 NalR ΔGifsy-1 ΔGifsy--2 ΔGifsy-3 ΔST64B | This study |
| 11160 | *S.* Typhimurium ATCC 14028 NalR ΔGifsy-1 | This study |
| 11162 | *S.* Typhimurium ATCC 14028 NalR ΔGifsy-2 | This study |
| 11224 | *S.* Typhimurium ATCC 14028 NalR ΔGifsy-3 | This study |
| 11958 | *S.* Typhimurium ATCC 14028 NalR Δ*xis*/*int* | This study |
| 11976 | *S.* Typhimurium ATCC 14028 NalR Δ*recET* | This study |
| 12074 | *S.* Typhimurium ATCC 14028 NalR Δ*SRRz* | This study |
|  |  |  |
| *Abbreviations: Nal, nalidixic acid; *kan*, kanamycin resistance; *cat*, chloramphenicol resistance. For the sake of brevity, the Gifsy-1 holin, endolysin, and spannin gene homologs are referred to in the text as *S*, *R*, and *Rz*, respectively, as in bacteriophage λ. The GenBank designations for the homologs in *S*. Typhimurium LT2 are STM2613.Gifsy1, STM2613.2n.Gifsy1, and STM2613.1n.Gifsy1, respectively. | | |

| **Table S2**: Primers used in this study for qRT-PCR | | |
| --- | --- | --- |
| Primer | Sequence | Target |
| 41A | TTATCTATCTGCGCAAGGGC | *sb41* [5] |
| 41B | CAGGTTGAGCGAGGGTTG | *sb41* [5] |
| Gif-1_For | TAATACCGCAATACCGTTCACTACCTG | STM2605 |
| Gif-1_Rev | CGAAAGTCTGGATCAGTCGGATATG | STM2605 |
| Gif-2_For | GTACTGTTGTCTCAGAGAATGTC | STM1048 |
| Gif-2_Rev | GTGATATCAAATTCTGTGTTCCAGATCC | STM1048 |
| Gif-3_For | TCTTAACTGAGCACGATATTCACCGCACC | *sspH1* |
| Gif-3_Rev | CTGACTGAAGAAGTCTCCCCTGTTTCACC | *sspH1* |
| trpA_For | GGGAAATCTGATGGAACGCTACGAA | *trpA* |
| trpA_Rev | TTTCAGTGACTGTTCAATGCCAGGG | *trpA* |
|  |  |  |

| **Table S3**: Primers used for generation of deletion mutants | | |
| --- | --- | --- |
| Primer | Sequence* | Target |
|  |  |  |
| k1 | CAGTCATAGCCGAATAGCCT | *kan*(*aph*) gene |
| k2 | CGGTGCCCTGAATGAACTGC | *kan*(*aph*) gene |
| kt | CGGCCACAGTCGATGAATCC | *kan*(*aph*) gene |
|  |  |  |
|  | Gifsy-1 gene deletions (pKD4 template): |  |
| G1RECEF | ACCAGATATATCGGGGTGCT | *recE* Gene |
| G1RECER | ATGGTGTTTGGTGCCTGCTG | *recE* Gene |
| G1RECTF | ACACTGGTGAATGGTCAGCG | *recT* Gene |
| G1RECTR | GCGATTTATTAGGCGCATAA | *recT* Gene |
| G1RECEH1P1 | GTGCTGTTAAGGCACCTCCATTCTACACGAATTGAGGACAAAACAATGTGTAGGCTGGAGCTGCTTCGA | *recET* Deletion |
| G1RECTH2P2 | ATAAAACCTCCTCAGGTGGGAGGGCGTACCCCCTCCCGATGCAATTAGCCCATATGAATATCCTCCTTAG | *recET* Deletion |
| G1XISF | TGCCAGCACAGGCAAACTGA | *xis* Gene |
| G1XISR | AAGCTTATAGTAAATGGTGG | *xis* Gene |
| G1INTF | TCCATATCCTATGATGGACA | *int* Gene |
| G1INTR | CACGACCAACACGCCGTCCA | *int* Gene |
| G1XISH1P1 | AAACTGAATATTAGCGATGGCCCGCTGCGGGGCCACTGGAGAAAACGATGTGTAGGCTGGAGCTGCTTCGA | *xis-int* Deletion |
| G1INTH1P1 | TGGACACAGAATCTTCCGTTCTGATGGACACATGCAGGGATAAATCATGCATATGAATATCCTCCTTAG | *xis-int* Deletion |
| G1SF | CCCAACCATTTCACTCACTC | S Gene |
| G1SR | GGGTCCGGATAAGCTTTCAG | S Gene |
| G1RzF | TACCCGTCGGCGTGAGGCGG | Rz Gene |
| G1RzR | GTTTATGCATTTCTGAAACT | Rz Gene |
| G1SH1P1 | CTCACTCTAGTTACCATCCGAAATCATCGGAGGTGAGGCTTATGAAAATGTGTAGGCTGGAGCTGCTTCGA | SRRz Deletion |
| G1RzH2P2 | CCCGCAGGTAAGCTCCTTTTCCCTCCTGCGGGAATTTTTTATTTGCACTGCATATGAATATCCTCCTTAG | SRRz Deletion |
|  |  |  |
|  | Gifsy-3 prophage deletion (pKD13 template): |  |
| pp599 | TTGGCGGTATCGGTATTG | *attL* Site |
| ppR25 | CGGTTCGCTTGCTGTCCATA | *attL* Site |
| pp587 | GCCTGCTTGCCGAATATC | *attR* Site |
| pp549 | CACGCCCGACATTATAAG | *attR* Site |
| ppAG72 | CCCGTTTTTATTACCTTCTTAAAGTTCTTCCCCAAAACTTTCCCCGATCCGTCGACCTGCAGTTC | Gifsy-3 Deletion |
| ppAG73 | GAGGGAGTTTTGATAAAGTTTTGATAACCGTTCGAATACTAATAATGTGTAGGCTGGAGCTGCTT | Gifsy-3 Deletion |
|  |  |  |

*Abbreviations: *kan*(*aph*), kanamycin resistance cassette of pKD4 or pKD13 template plasmids; *attL*, *attR*; Gifsy-3 left and right attachment/integration site sequences in the chromosome. Sequences for screening of Gifsy-1 gene deletions were based on sequences available from the Stanley Falkow laboratory at <https://falkow.stanford.edu/whatwedo/wanner/>. Sequences for the common priming sites for amplification of the kanamycin resistance cassettes in pKD4 or pKD13 and internal kanamycin resistance gene (k1, k2, kt) are from Datsenko and Wanner (2000). The mutagenesis primers for amplification of the antibiotic cassettes of either pKD4 (Gifsy-1 gene deletions) or pKD13 (Gifsy-3 deletion) are based on published genome sequences for *S*. Typhimurium LT2 and ATCC 14028, respectively.

**References**

1. Stanley, T.L., C.D. Ellermeier, and J.M. Slauch, *Tissue-specific gene expression identifies a gene in the lysogenic phage Gifsy-1 that affects Salmonella enterica serovar typhimurium survival in Peyer's patches.* J Bacteriol, 2000. **182**(16): p. 4406-13.

2. Ho, T.D., et al., *Identification of GtgE, a novel virulence factor encoded on the Gifsy-2 bacteriophage of Salmonella enterica serovar Typhimurium.* J Bacteriol, 2002. **184**(19): p. 5234-9.

3. Figueroa-Bossi, N. and L. Bossi, *Resuscitation of a defective prophage in Salmonella cocultures.* J Bacteriol, 2004. **186**(12): p. 4038-41.

4. Braetz, S., et al., *The role of ATP pools in persister cell formation in (fluoro)quinolone-susceptible and -resistant strains of Salmonella enterica ser. Typhimurium.* Vet Microbiol, 2017. **210**: p. 116-123.

5. Alonso, A., et al., *Increased excision of the Salmonella prophage ST64B caused by a deficiency in Dam methylase.* J Bacteriol, 2005. **187**(23): p. 7901-11.

6. Datsenko, K.A. and B.L. Wanner, One-step inactivation of chromosomal genes in Escherichia coli K-12 using PCR products. Proc Natl Acad Sci U S A, 2000. **97**(12): p. 6640-5.
